# Quackery

**Published:** 1844-06

**Authors:** 


### ARTICLE X.

### *Quackery.*

This term has usually been restricted to the professions of divinity, medicine and law, but we find by tracing it more narrowly, that it reaches every art, trade and occupation, and in a still more enlarged and comprehensive sense, that it embraces the whole being of man, as comprised under that of his physical, intellectual, and moral nature.

Hypocrisy, imposture, bold, daring, unblushing pretensions, without any just claims—mid-day robbery on the temple of science, by stealing her mantle and wearing it without license—*modestly* telling the people at the same time, in trumpet tongue, that it is truly genuine, and demonstrating thereby, most infallibly, that its rougish possessor has all the popular essentials of surpassing skill and success.

In a word, all shallow and clamorous pretensions, under whatever name they may appear, and however plausible they may seem, come strictly under the head of quackery—a term expressive on the one hand, of complete destitution—an entire waste, in all the essentials of worth in reference to qualifications for practice in the particular profession or branch of business pursued—and on the other, denoting the positive possession of an exterior covering to deceive the credulous,—being as it is, a most glossy effrontery, assurance and charlatanism, thereby committing a more unpardonable outrage on all the sacred rights of humanity, truth and decency.

Intellect, morals and religion, form the three-fold cord—the only solid foundation, from and on which all that is really useful to mankind must be drawn and reared—and by which, also, every thing that pretends to be useful and good, must be infallibly tested and exposed.

This, in a word, is the rock of truth—whether in science or art—every thing, therefore, coming in collision with this touchstone, if true, will naturally glide into and become part and parcel of this fundamental rock of truth—being as it is of the same essential nature; while on the other hand, all that is false, when coming in contact with it, will be as certainly repulsed and dashed to pieces—for having no affinity with it—no right to any part or parcel in this rock of blessings, but doomed as an outcast from this holy habitation, and condemned, yea, justly condemned, both as the enemy of truth and mankind.

This *rock* we have said, is constituted of *intellect, morals* and *religion.*

The three go hand in hand—they are inseparably and harmoniously blended—though it may be in different degrees—but the whole, nevertheless, forming the most perfect unity in the individual who has reduced them to practical cultivation—and it is the disjunction of one or more of these essential elements—the taking of one and rejecting the other, instead of taking the whole as the absolute and indispensable condition; we say, we believe this to be the true source of all the quackery in the land; for, intellect grasps at the facts before it, weighs them carefully in the scales of the judgment, and thus acquaints itself with their applicability and utility. Morals, or the duty we owe to our fellow men, point out the road that intellect should take in the practical application of its knowledge, which is the benefit of mankind—

while religion comes in, backs both intellect and morals—binds the two more strongly together, and thus form, by their conjoint action, that rock of truth which has the good of mankind, individually and collectively, and good only in its most sacred keeping and dispensing. While if intellect *alone* pretends to be the whole truth and dispenses as such, leaving out morals and religion, we find self the predominant thing, and the good of mankind entirely lost sight of,—and we have thus formed a quack, who, by being destitute of moral principle, is only rendered by his shrewdness and intelligence a still more dangerous person in society, and still more capable of gulling the credulous public.

Again, persons the most strictly moral and religious may be, and, we are sorry to have to add, daily are the vilest quacks on earth. Many of our ministers, not to mention others in inferior stations, practice in things—medicine for example—of which they are not only wholly ignorant, but lend their influence to others to do the same thing, who are equally ignorant—thus committing the two-fold (though we trust, unintentional) crime, of first quacking themselves, and then helping to manufacture others—from simply, though very benevolently, assuming that morals and religion *alone,* are sufficient without intellect, or proper information on the subject. This is a mistake, we believe, most alarmingly extensive, and strongly demanding a speedy corrective.

The moral and physical laws, though they may and do act in harmony, are nevertheless independent the one of the other. A physical law declares a vessel will sink, if she spring a leak, or her bottom start, and all on board perish, let their morality and religion be ever so pure.

So, a patient may and will as certainly perish, if he take a dose of poison, ignorantly administered, though it be by the best of divines—for nature asserts that a physical law has been most grossly violated—and notwithstanding the moral and divine law may be thoroughly kept, yet the two sets of laws are so far disconnected and independent, the one of the other, as not to be able to avert the penalty of one by keeping the other, but that each will have its separate reward and punishment for each separate violation and obedience. With these remarks on quackery in general, we propose to offer a few suggestions, in continuation of those we made in the last number of our paper, on that species of imposture denominated Dental Quackery—and

First: We would suggest that every state in the Union should follow the noble example set by Maryland in declaring, through her legislature, that it is high time this much neglected branch of science should be more attended to and cultivated—that its principles should be more extended and understood—and finally, that the public should be more protected than it has hitherto been, by the lights of dental science, from the daily presumption and ignorance of such hosts of practising pretenders.

Hence the legislature of Maryland, in addition to expressing her sense of the suffering and cruelty which the people were enduring from allowing such an immense number of ignorant pretenders to stalk abroad, and freely and fearfully to practice upon the health and lives of the community, without scarcely a ray of light from the sun of dental science to guide through, and help to stay and dispense this midnight gloom.

We say, in such condition of mental darkness and practice, the legislature of Maryland saw fit to incorporate the Baltimore College of Dental Surgery, as one means of dispensing light and knowledge on this subject, and thus furnish suitable persons who will go from this highly important institution, thoroughly qualified to practice properly the art of dentistry, expose the impositions of quacks, and thus successfully shield an important interest of society.

The great and prime object of this College is to elevate the standard and give dignity to the dental profession—by giving its pupils a sound and thorough education, both in the principles and practice of dentistry.

As it may not be generally known, it may be well to mention its general scope of instruction. There are four professorships, viz.

1. Dental Physiology and Pathology.
2. Practical Dentistry.
3. Special Pathology and Therapeutics.
4. Anatomy and Physiology.

And here we would wish to call special attention to the fact of there being a chair expressly provided for teaching the practical parts—embracing all the manipulations pertaining both to the surgical and mechanical departments of the profession.

In a word, preparing the student in a more thorough manner, and in a much shorter time than can possibly be done in any dentist's office for the immediate and active duties of his profession—while at the same time he has the advantage of being carefully taught the principles of the science—not simply the healthy and morbid conditions of the teeth—but further, the various relations of the teeth in these two states to the whole and every part of the body.

Hence, disease, general and special—and anatomy, general and special, from being related with, and especially influenced by, the condition of the dental apparatus, are considered as essential parts of instruction to a properly qualified dentist.

A second suggestion is, that persons about to enter the dental profession, should secure proper preparatory educations before entering it.

This would be a most important step to banish quackery, for when the mind becomes enlightened even in general knowledge, it will most usually, unless those roughly corrupted, shrink from practising any particular portion of knowledge—without suitable qualifications.

And the last suggestion we at this time have to offer, is the necessity of union among the dentists throughout the whole United States—union of feeling and interest—a reciprocal and willing interchange of opinion and practice, and frequent meetings and associations, for the diffusion of dental light and truth among the profession in particular, and the people at large—as another and powerful means of staying the torrent of dental quackery which is still sweeping over our land.—*Dental Intelligencer.*

